# Active On-Chip Dispersion Control Using a Tunable Silicon Bragg Grating

**DOI:** 10.3390/mi10090569

**Published:** 2019-08-28

**Authors:** Charalambos Klitis, Marc Sorel, Michael J. Strain

**Affiliations:** 1 School of Engineering, University of Glasgow, Glasgow G12 8LT, UK; 2 Institute of Photonics, Department of Physics, University of Strathclyde, Glasgow G1 1RD, UK

**Keywords:** silicon photonics, dispersion control, Bragg gratings

## Abstract

Actively controllable dispersion in on-chip photonic devices is challenging to implement compared with free space optical components where mechanical degrees of freedom can be employed. Here, we present a method by which continuously tunable group delay control is achieved by modulating the refractive index profile of a silicon Bragg grating using thermo-optic effects. A simple thermal heater element is used to create tunable thermal gradients along the grating length, inducing chirped group delay profiles. Both effective blue and red chirp are realised using a single on-chip device over nanometre scale bandwidths. Group delay slopes are continuously tunable over a few ps/nm range from red to blue chirp, compatible with on-chip dispersion compensation for telecommunications picosecond pulse systems.

## 1. Introduction

Integrated Bragg grating filters are an established and widely used technology on the Silicon-on-Insultor (SOI) material platform. They are applied in a large variety of applications, including, optical filtering [1,2], sensing [3], laser cavity feedback [4] and all-optical signal processing [5,6]. A wide range of device geometries have been demonstrated in order to exercise control over the grating optical characteristics, namely the filter bandwidth [7], ripple [8], extinction and dispersion [2,9,10]. In turn, the optical characteristics of the grating can be designed through the coupling coefficient, κ, and the grating Bragg wavelength, $\lambda_{B}$, as a function of the propagation length [10,11]. Active control of Bragg grating devices has also been demonstrated using multi-section p-n junction elements to create tunable notch features in the device stopband [12].

The control afforded by the Bragg grating device over the dispersion of a propagating signal is crucial in applications such as all-optical signal processing [5] and pulse shaping [13]. Commonly, the group delay profile of a device is varied through the local Bragg wavelength function. Therefore, by simply varying the grating period [14], or effective waveguide index, devices can be created with linear and higher order dispersion profiles [9] or even complex multi-band filter designs [15]. Filter ripple effects have been effectively minimised by suitably apodising the grating κ along its length [9,10,16]. The dispersion profile of a grating device is generally defined during the fabrication procedure as a modulation of the period [14] or effective index [17]. Some rigid tunability of the device spectrum has been demonstrated through global thermal [18] or electronic tuning [19]. Narrowband delay has also been demonstrated using multi-section gratings at the band-edge [20,21]. However, for future Photonic Integrated Circuits (PICs), true tunable dispersion on-chip will be a valuable component functionality. The principle of reconfigurable silicon photonic circuits has been well established in devices including ring resonators [22], Mach–Zehnder interferometers [23,24], and modulators [25], using both thermo-optic and carrier injection methods.

In this work, we present the design considerations for realising tunable dispersion gratings on the silicon photonics platform and present measured performance of fabricated devices. Control is demonstrated producing both red and blue chirp in a single device over bandwidths and group delay ranges compatible with picosecond pulse compensation schemes.

## 2. Results

### 2.1. Actively Tunable Bragg Grating Design

**Figure 1:**
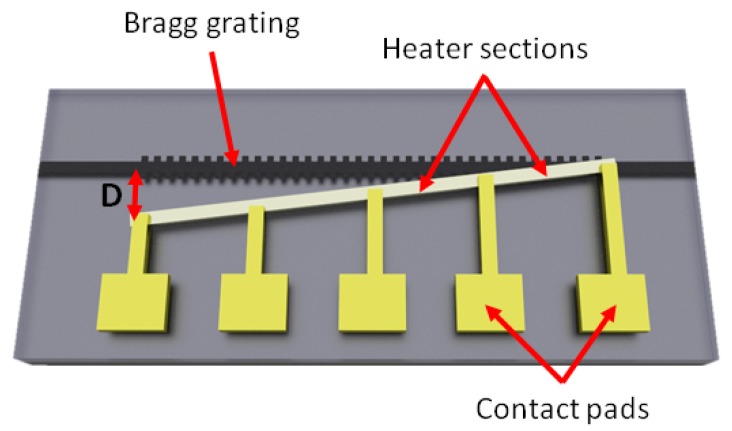
Schematic of a Bragg grating with an integrated heater element. The displacement between the waveguide axis and heater central position is a function of propagation length, with a maximum displacement *D*.

The Bragg grating device is implemented with a constant Bragg wavelength, $\lambda_{B}\left(z\right)$, along its length. To create a chirp in the grating response, the Bragg wavelength function is controlled by creating a thermal gradient along the device length. A schematic of the device is shown in Figure 1.

**Figure 2:**
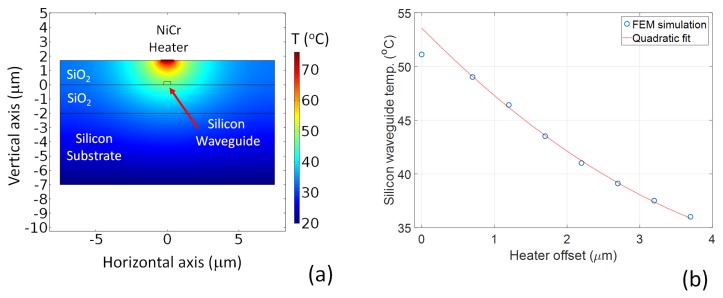
(**a**) FEM thermal model of a heater element co-axial with a silicon-on-insulator waveguide; and (**b**) calculated temperature of the silicon waveguide as a function of offset of the heater central axis from the central axis of the waveguide.

It has been demonstrated by a number of groups that by placing a resistive element over a waveguide, the local index of refraction can be tuned through the thermo-optic effect [26,27,28]. Furthermore, due to the large mismatch in thermal conductivity of silicon and silica, the heat dissipation as a function of lateral displacement from the element allows differential heating of closely spaced structures, e.g., for tuning the coupling fraction of an evanescent field coupler [29]. Thus, by making the heater element displacement from the waveguide a function of the propagation length, local variations in refractive index, and hence Bragg wavelength, can be induced in the grating. Figure 2a shows a cross-sectional, thermal finite element model of the heater element above a silicon-on-insulator waveguide in the steady state. By varying the offset between the central axis of the heater element and the silicon waveguide, the temperature of the silicon guide can be controlled. In this example, the heater element is dissipating 11 mW of power. For power dissipation levels similar to the results presented here, the thermal control element has a temperature of tens of degrees above room temperature. The heater element then dominates the thermal state of the silicon waveguide with respect to environmental temperature variations. No active temperature stabilisation of the silicon substrate was used in this work. Figure 2b shows the relationship between the offset of the heater axis to the waveguide and the resultant waveguide temperature. The curve fits well to a quadratic function. There is a variation from the curve when the waveguide and heater are co-linear. This is due to the finite nature of the heater width, resulting in saturation of the temperature as a reducing function of offset. The thermal impedance of the silica layers also results in a high thermal gradient over micron scales, and therefore allows longitudinal chirping of the grating structure. Thus, given the quadratic relationship between offset and waveguide temperature, and the proportional relationship between temperature and material refractive index, a refractive index chirp function can be designed as a function of heater displacement from the waveguide axis.

In Figure 1, a signal injected from the left hand side of the device would see an increasing refractive index with propagation length through the grating, and therefore an effective red chirp. Alternatively, light coupled in from the right hand side of the device would see decreasing refractive index with propagation length and, therefore, effective blue chirp. The magnitude of the induced chirp is dependent on the grating length, coupling coefficient and thermal gradient. The difference in temperature and, therefore, refractive index, along the grating can be controlled electronically through the power dissipated in the heater.

**Figure 3:**
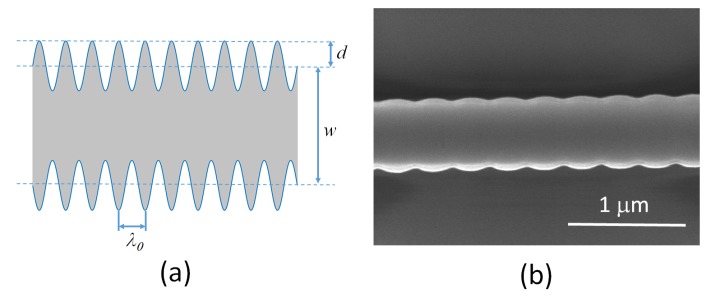
(**a**) Schematic of a sinusoidal sidewall perturbation Bragg grating, and (**b**) SEM image of a fabricated device.

To demonstrate the tunable dispersion concept, a grating response was chosen to cover bandwidths of ≈1 nm, and group delays in the picosecond range, corresponding to typical dispersion compensation requirements for picosecond telecommunications pulses. The gratings were designed on a silicon-on-insulator platform with a core layer thickness of 220nm and a mean waveguide width of 500nm. The grating period, $\Lambda_{0}$, was set at 318 nm, to give a Bragg wavelength around 1550nm. The grating was designed with a length of 250μm and a sinusoidally varying sidewall amplitude modulation, *d*, of 6nm. The grating perturbation is realised as a sinusoidal variation around an average waveguide width, *w*. A schematic of the sidewall perturbation grating is shown in Figure 3 along with a SEM image of the fabricated device before deposition of the upper-cladding.

A Gaussian apodisation of the sidewall perturbation was applied to minimise ripples in the grating amplitude and group delay response. The grating response can simulated using a Transfer Matrix Method (TMM) model with $\lambda_{B}\left(z\right)$ as a parameter [30]. Since $\lambda_{B}\left(z\right)=2n_{\mathrm{eff}}\left(z\right)\Lambda_{0}$, where $n_{\mathrm{eff}}\left(z\right)$ is the local modal refractive index and $\Lambda_{0}$ is the grating period, the grating chirp can be modelled using a variation in refractive index. The modal index is in turn dependent on the temperature at the waveguide generated by the local heater element. The heater element is fabricated using a 900nm wide track of 50nm thick NiCr. To create a linear chirp profile, the displacement, D(z), between the centre lines of the waveguide and heater element, is varied using a $z^{0.5}$ relationship. It is worth noting that the waveguide dispersion of silicon nanowire waveguides is significant [31], and therefore must be taken into consideration when designing and modelling Bragg gratings in silicon.

**Figure 4:**
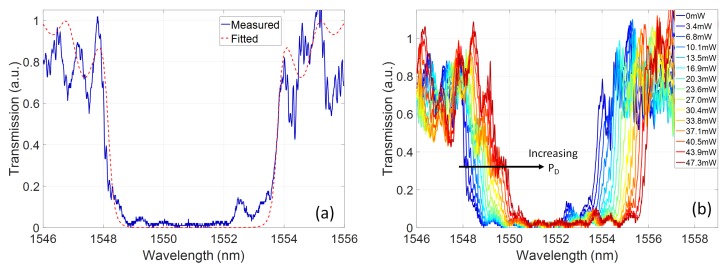
(**a**) Measured and fitted Bragg grating transmission spectra of the grating device in the unchirped state; and (**b**) measured grating spectra for increasing power dissipation, P${}_{D}$, on the thermal heating element.

Figure 4a shows a measured transmission spectrum of the fabricated device with no power dissipated on the heater, i.e., the device is unchirped. Measured device spectra can be fitted to the TMM model using a least squares curve fitting method. In this case, the form of the grating chirp is assumed, e.g., unchirped or linear blue-chirp. The coupling coefficient and the maximum and minimum effective index values at the grating ends are then left as free parameters to perform the fit. The extracted modal index from the fitting of the unchirped grating is 2.4386 and the coupling coefficient is 323cm${}^{-1}$. Furthermore, since an apodisation scheme based on variation of the grating sidewall amplitude was employed, residual ripples in the grating reflectivity and group delay spectra are still apparent. Grating coupling coefficient apodisation schemes that more effectively reduce the spectral ripple of the device, and can be fabricated within nanometric tolerances, have recently been demonstrated using phase based methods [10,16].

**Figure 5:**
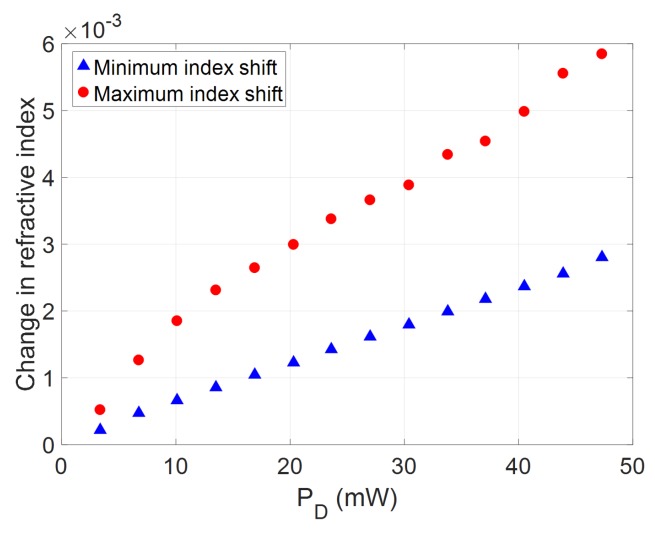
Variation in minimum and maximum thermally induced modal refractive index shifts of the device extracted from fitting of the transmission spectra.

To assess the effects of the heater on the z-dependent modal index, the grating transmission spectrum was measured as a function of total electrical power dissipated on the heater element, $P_{D}$. Figure 4b shows the measured transmission spectra, linearly varying $P_{D}$ up to 47.3mW. The grating fitting was carried out in each case, assuming a linearly varying modal index as a function of the propagation length, with extreme modal index values as free parameters. The local shift of the Bragg grating along the device can be decomposed into two components: a baseline shift of the full device since the full length sees some minimum thermal increase, and a relative temperature change along the propagation direction due to the displacement of the heater section. This can be approximated as:

(1) $$\lambda_{B}\left(z\right)=2\left(n_{\mathrm{eff},0}+dn_{\min}+dn\left(z\right)\right)\Lambda_{0}$$

where $n_{\mathrm{eff},0}$ is the effective modal index of the grating waveguide at room temperature, $dn_{\min}$ is the increase in modal effective index at point of greatest displacement between the waveguide and the heater element, and dn(z) is the additional thermal increase along the grating length above $dn_{\min}$ as the displacement of the heater and waveguide reduces. Figure 5 shows the extracted values of $dn_{\min}$ and $dn{\left(z\right)}_{\max}$ from the transmission spectra. As s expected, $dn{\left(z\right)}_{\max}$ has a higher gradient as a function of $P_{D}$ than $dn_{\min}$, indicating the effective chirping of the grating response.

### 2.2. Group Delay Chirp Control

**Figure 6:**
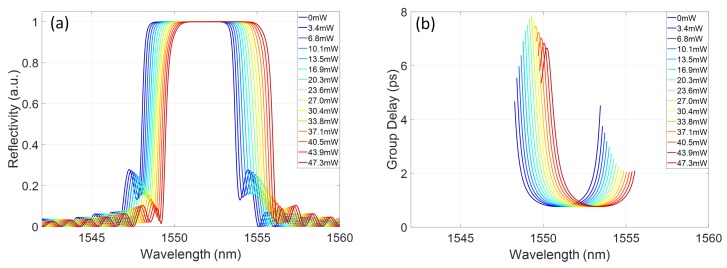
(**a**) Simulated reflectivity; and (**b**) group delay of a Bragg grating device with thermally induced refractive index differences as parameters.

Figure 6 shows the calculated reflectivity spectra and group delay profiles for the grating, with the extracted values of $dn_{\min}$ and $dn{\left(z\right)}_{\max}$ as parameters. The group delay is only calculated for the portion of the reflectivity spectrum above 90% of its maximum value.

**Figure 7:**
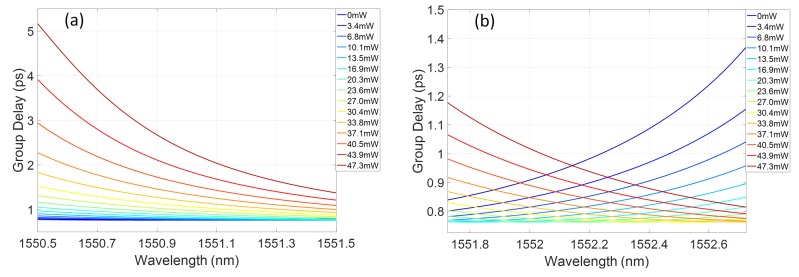
Simulated group delay bands as a function of dissipated power on the heater centred at: (**a**) 1551nm; and (**b**) 1552.2mn.

The group delay profile of the grating response shows a sensitivity to the induced refractive index profile, with an increasing blue chirp developing across the central region of the grating reflectivity band. The wide stopband, due to the high grating coupling coefficient, means that, even for chirped gratings, there is a portion of the reflection band with flat group delay. As noted previously, 1 nm bandwidths are considered here, corresponding to typical telecommunications picosecond pulse sources. By selecting a particular central wavelength within the grating stopband, controllable chirp can be exhibited as a function of power dissipated on the heater. For example, sub-bands centred at 1551nm and 1552.2nm are shown in Figure 7a,b, respectively. In Figure 7a, increasing the dissipated power on the heater induces a variation in the blue chirped gradient of the group delay in the few ps/nm range. Alternatively, by selecting a central wavelength towards the red tuned side of the grating reflection spectrum, a range of chirp values spanning from blue to red chirp can be accessed, as shown in Figure 7b. As this change from blue to red chirp crosses a zero dispersion condition, the absolute chirp rate is less than the blue chirp rate achievable at similar power dissipation levels, at bands further detuned from this wavelength.

**Figure 8:**
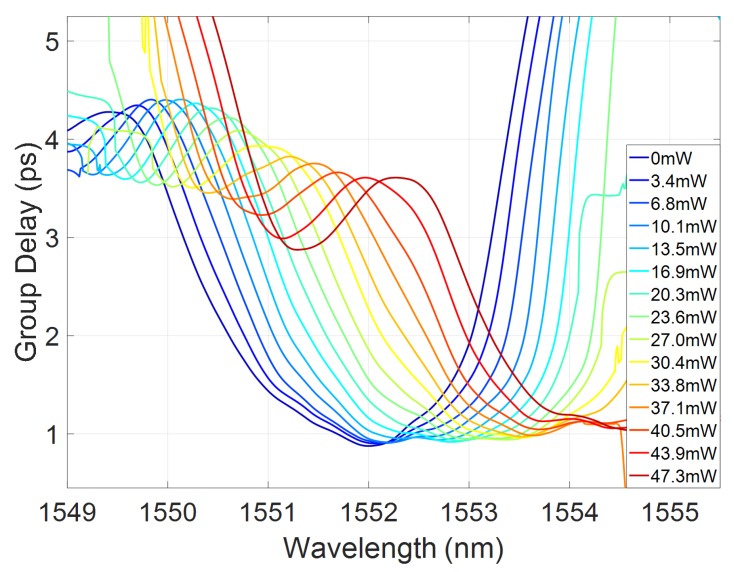
Measured group delay tuning as a function of dissipated power on the heater.

The group delay of the fabricated tunable grating was measured as detailed in Section 5. Figure 8 shows the measured group delay as a function of power dissipated on the heater.

**Figure 9:**
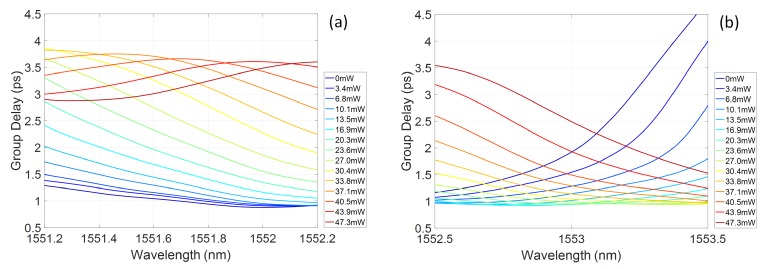
Measured group delay bands as a function of dissipated power on the heater centred at: (**a**) 1551.7 nm; and (**b**) 1553mn.

As expected, there is a clear increase of the group delay in the centre of the band, indicating increasing blue chirp of the spectrum. Evidence of the residual ripple in the grating reflectivity is also apparent in the group delay kink developing for increasing dispersion. Figure 9a,b shows sub-bands of the grating response centred around 1551.7 and 1553nm, respectively. As predicted, for the lower wavelength range, increasing the heater power creates an increasing group delay slope, with effective blue chirp. In the longer wavelength band, both blue and red chirp responses can be accessed, passing through a flat group delay characteristic.

**Figure 10:**
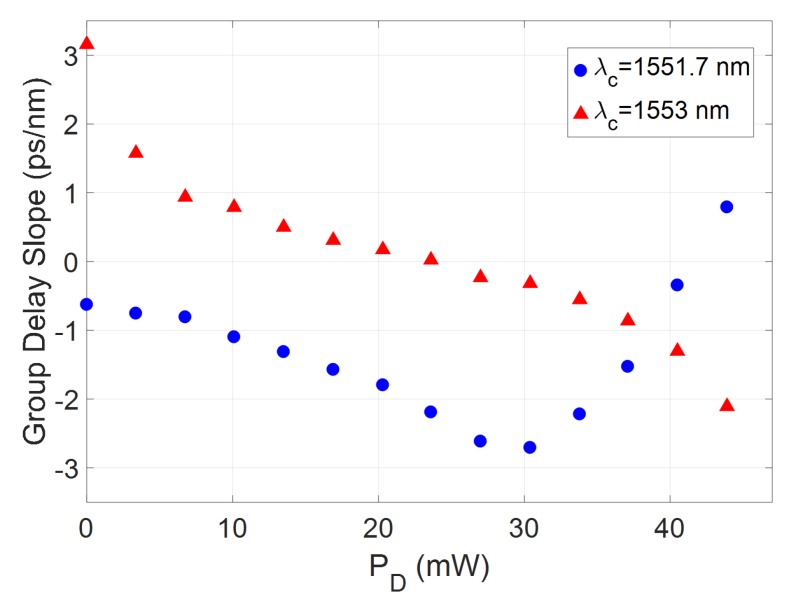
Measured group delay slope as a function of dissipated power on the heater corresponding to the dispersion bands centred at 1551.7 nm; and 1553nm.

Figure 10 presents the average group delay slope for both sub-bands in Figure 9. In both cases, quasi-continuous tuning of the group delay slope is demonstrated in the ps/nm range, with both blue and red chirp accessible from a single device and from a single injection direction.

## 3. Discussion

**Figure 11:**
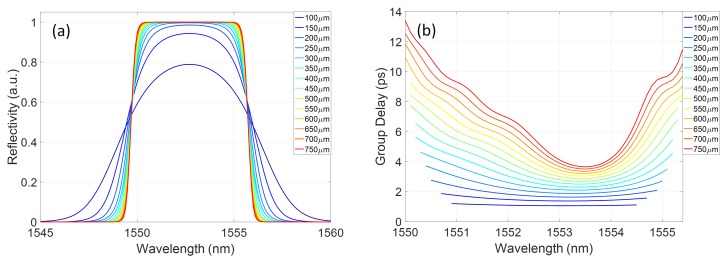
Simulated grating: (**a**) reflectivity; and (**b**) group delay spectra with grating length as a parameter.

By integrating a thermal heater element with a varying displacement along the length of a uniform Bragg grating device, thermal gradients and therefore local modal refractive index profiles have been imposed onto the device. The strong grating coupling coefficient means that the full group delay profile is complex, with both blue and red chirp regions occurring over a bandwidth of a few nanometres. However, this can be used to sub-select regions of interest for dispersion control over nanometre scale bands, suitable for picosecond pulse systems. In this way, both blue and red chirp control have been demonstrated in a single device. The group delay slope is also related to the grating length, i.e., longer physical distances between grating sections tuned to different spectral components will induce larger group delay shifts between those spectral components [32]. Therefore, to tailor the accessible maximum group delay slope, the grating length can be defined as a design parameter. Figure 11 shows TMM simulated grating reflectivity and group delay spectra, dependent on the grating length. In these simulations, the thermal index gradient was assumed to be linear and corresponding to the measurement conditions for the maximum power dissipated on the thermal heater of 47.3mW. The apodisation profile is Gaussian.

The group delay range across the spectrum is, as expected, dependent on the grating length. Therefore, devices can be designed based on the required application requirements.

In addition to the relative group delay dispersion induced across the device, the absolute spectral position of the grating response is dependent on two main factors. The first is related to fabrication tolerances in the manufacture of the device. Since the absolute Bragg wavelength of a fixed period grating is proportional to its effective modal index, any variation from the design geometry will lead to a shift in the resultant Bragg spectrum. However, in high resolution fabrication schemes such as optimised electron beam or deep ultraviolet (UV) lithography, such variations are global rather than local. That is, the average waveguide width may vary from run to run, or even between spatially separated devices, but is unlikely to see significant variation across a single device. Typical geometrical variations can be in the nanometre range, leading to effective index variations in the order of $10^{-3}$–$10^{-2}$ [33]. The second factor affecting the absolute spectral position of the Bragg grating response is the global thermal shift experienced by the full grating when the heater is electrically driven. The thermal element induces both a global wavelength shift of the grating characteristic and a local gradient to induce the chirped profile, as detailed in Figure 5, The global, or minimum index, shift experienced by the full grating is in the order of $10^{-3}$, comparable with any potential geometric effects. To implement control over both the group delay slope and central wavelength, a second heater element could be implemented parallel to the Bragg grating. This element would produce a rigid index shift of the full grating response, shifting the demonstrated group delay curves in wavelength. Therefore, using both heater controls, varying group delay slopes in both chirp directions could be accessed at a single central wavelength, as long as the un-heated grating central Bragg wavelength was designed to be at a shorter wavelength than the required application space.

## 4. Conclusions

Control over the group delay slope of an integrated silicon Bragg grating is achieved using a simple thermal tuning element. Spatial displacement of the heater along the propagation length of the grating produces a longitudinal variation in modal effective index, and, therefore, dispersion. Variation of the electrical power dissipated by the heater allows for control over the group delay slope, with fabricated devices covering the range of 0–3 ps/nm over nanometre range bandwidths. This range is ultimately limited by the grating length and can be designed for specific applications before fabrication. Finally, due to the strong coupling coefficient of the gratings presented in this work, both blue and red chirp have been demonstrated in a single device, with the possibility to tune across this full range for a particular central wavelength condition.

## 5. Materials and Methods

### 5.1. Device Fabrication

The devices were fabricated on a 220 nm thick silicon core on a buried oxide lower cladding layer of 2μm. The patterns were written using e-beam lithography into hydrogen silsesquioxane (HSQ) resist that was subsequently used as a hardmask for Inductively Coupled Plasma (ICP)-Reactive Ion Etching (RIE) etching of the silicon. Polymer spot-size convertors [34] were fabricated to match the input lensed fibre mode to the waveguides. The final devices were coated with a 900nm thick silica uppercladding before definition of the heater elements and electrical transmission lines. The heaters were fabricated as 900nm wide strips of 50nm thick NiCr. Multiple point contacts on the heater lines were defined to reduce the necessary voltage required to drive the device, as shown in the schematic of Figure 1. The heaters were divided into 50μm long sections, each with a resistance of ≈1.5 kΩ, and were driven in an alternating positive/ground, arrangement.

### 5.2. Transmission and Group Delay Measurements

**Figure 12:**
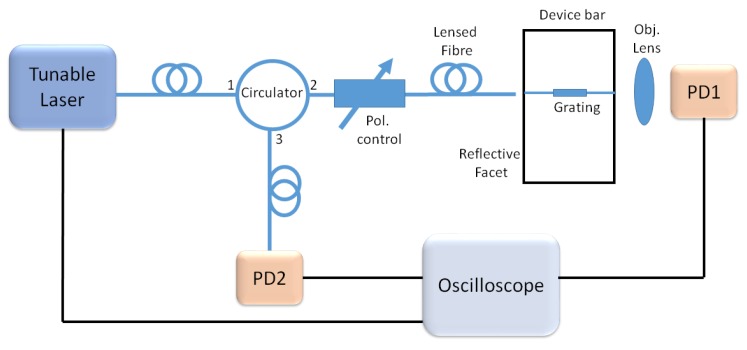
Transmission and group delay measurement setup. (Pol., polarisation; PD, photodiode).

The Bragg grating devices were probed using a tunable laser coupled through fibre polarisation optics and a lensed fibre tip to the chip, as shown in Figure 12.

**Figure 13:**
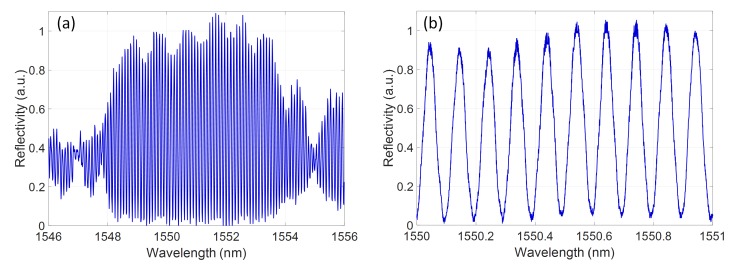
(**a**) Measured reflectivity spectrum of the grating with no induced chirp; and (**b**) detailed spectrum of the Fabry–Perot fringes created by the intereference between the device bar facet and grating reflector.

The transmission spectra were measured by coupling the light from the output facet of the device bar to an objective lens and InGaAs photodiode. The reflected spectra were captured by coupling the back reflected light from the chip through a circulator to an InGaAs photodiode. Both photodiode signals were measured using an oscilloscope, triggered from the swept tunable laser source. The reflected spectra exhibit Fabry–Perot interference fringes created by the cavity formed between the reflective facet of the device bar and the Bragg grating, as shown in Figure 13. While the device bar facet is a broadband reflector with a spatially fixed position, the grating group delay profile creates a reflector with an effective spatial position that is wavelength dependent. Therefore, the effective Fabry–Perot cavity length is wavelength dependent and will create interference fringes that are directly related to the group delay response of the grating. This interference allows the direct extraction of the Fabry–Perot cavity phase, and hence group delay, introduced by the grating element as detailed in [35]. Figure 8 shows the measured group delay curves for the device measured using this Fabry–Perot technique.
